# Simple, Rapid and Inexpensive Quantitative Fluorescent PCR Method for Detection of Microdeletion and Microduplication Syndromes

**DOI:** 10.1371/journal.pone.0061328

**Published:** 2013-04-19

**Authors:** Martin Stofanko, Higgor Gonçalves-Dornelas, Pricila Silva Cunha, Heloísa B. Pena, Angela M. Vianna-Morgante, Sérgio Danilo Junho Pena

**Affiliations:** 1 GENE - Núcleo de Genética Médica, Belo Horizonte, Minas Gerais, Brazil; 2 Departmento de Bioquímica e Imunologia, Instituto de Ciências Biológicas, Universidade Federal de Minas Gerais, Belo Horizonte, Minas Gerais, Brazil; 3 Departamento de Genética e Biologia Evolutiva, Instituto de Biociências Universidade de São Paulo, São Paulo, São Paulo, Brazil; University of Bonn, Institut of Experimental Hematology and Transfusion Medicine, Germany

## Abstract

Because of economic limitations, the cost-effective diagnosis of patients affected with rare microdeletion or microduplication syndromes is a challenge in developing countries. Here we report a sensitive, rapid, and affordable detection method that we have called Microdeletion/Microduplication Quantitative Fluorescent PCR (MQF-PCR). Our procedure is based on the finding of genomic regions with high homology to segments of the critical microdeletion/microduplication region. PCR amplification of both using the same primer pair, establishes competitive kinetics and relative quantification of amplicons, as happens in microsatellite-based Quantitative Fluorescence PCR. We used patients with two common microdeletion syndromes, the Williams-Beuren syndrome (7q11.23 microdeletion) and the 22q11.2 microdeletion syndromes and discovered that MQF-PCR could detect both with 100% sensitivity and 100% specificity. Additionally, we demonstrated that the same principle could be reliably used for detection of microduplication syndromes, by using patients with the Lubs (*MECP2* duplication) syndrome and the 17q11.2 microduplication involving the *NF1* gene. We propose that MQF-PCR is a useful procedure for laboratory confirmation of the clinical diagnosis of microdeletion/microduplication syndromes, ideally suited for use in developing countries, but having general applicability as well.

## Introduction

Microdeletion and microduplication syndromes comprise a large group of human diseases that arise from imbalance in the transcription of genes due to partial loss or gain of genetic material of, typically, less than 5 Mb [1]. Symptoms range widely, but frequently encompass mental retardation, autism, physical dysmorphism and/or organ malformations.

Many microdeletion or microduplication syndromes can be diagnosed on the basis of phenotype alone, but molecular confirmation is essential for correct and reliable clinical and genetic prognoses. For that, a variety of techniques can be used, ranging from microscopic methods, such as Fluorescence *in situ* Hybridization (FISH), to molecular methods involving PCR and genome-wide microarrays [2], [3].

In a broad sense, laboratory diagnostic strategies can be classified as non-targeted or targeted. The most important non-targeted approaches are Array Comparative Genomic Hybridization (aCGH) and Single Nucleotide Polymorphism (SNP) arrays. The former has become the method of choice for diagnosis of microdeletions and microduplications in the US and Europe, while the latter provide a higher sensitivity to detect mosaic aneuploidies and is also capable of simultaneously diagnosing uniparental disomies and consanguinity [4]–[6]. Unfortunately, the very high cost of these analyses makes these methods inappropriate for the public healthcare system in developing countries.

Well-known targeted diagnostic approaches include FISH, Real-Time PCR and Multiplex Ligation-dependent Probe Amplification (MLPA) [2], [3], [7]. FISH is a complex and time-consuming method that needs special equipment, has limited resolution and is not appropriate for diagnosis of some microduplications [8], [9]. Quantitative Real-Time PCR discriminates between normal and affected individuals by differential amplification of target sequence due to different amounts of starting template DNA between control and deleted region. It offers fast turnaround time and technical feasibility, but it has limited multiplexing capabilities and suffers from reproducibility issues [10], [11]. In MLPA, a combination of probe hybridization and PCR followed by capillary electrophoresis and fluorescence quantification is used [12]. Its main drawback is dependence on sample DNA of high quality and lengthy and labor-intensive protocols [12], [13]. We here describe a simple, rapid and inexpensive quantitative fluorescent PCR method for targeted diagnosis of microdeletion/microduplication syndromes easily accessible for low-budget laboratories in developing countries.

All diagnostic methods based on quantitative comparison between amplification of target and control fragments using two different primer sets share a common constraint, the inherently unreliable amplification of distinct PCR fragments. This happens because PCR amplification is a complex exponential phenomenon and small differences in initial conditions may have a profound impact on the amounts of product obtained. To overcome this limitation, in 1991, Mutter and Pomponio reported on a simple PCR-based molecular technique that reliably permitted the diagnosis of dosage alterations of the human X and Y chromosomes [14]. They made use of differences in the sequences of the *ZFY* gene on the Y chromosome and its homolog *ZFX* on the X chromosome to design a single primer pair that led to the amplification of both genes. They showed that under these internal genomic competitive conditions it was possible to diagnose reliably individuals with 47,XXY or 47,XYY since the relative amounts of *ZFX* and *ZFY* products (differentiated by restriction analysis) were in 2∶1 and 1∶2 ratios, respectively, in contrast with a 1∶1 ratio for normal controls. This internally controlled means of establishing gene dosage in diagnosing human trisomy was further explored by Mansfield [15] who first utilized human polymorphic microsatellites in quantitative fluorescent PCR (QF-PCR), a method that has become widely used for the prenatal diagnosis of several human aneuploidies [16], and later by Lee et al. [17] who pioneered the use of simultaneous amplification of homologous genes in a method that they called HGQ-PCR. Both methods could reliably quantify amplicons of different sizes and thus determine chromosome dosage. Later, the attractive rationale of the HGQ-PCR method was explored, with technical variations, by Deutsch et al. [18] and Armour et al. [19] in detection of aneuploidy and genes with high copy number variation, respectively.

We show that the principle of the HGQ-PCR method of Lee et al. [17] can be used as a simple, rapid, DNA quality independent and internally controlled PCR-based detection test for sub-microscopic genome rearrangements that we have called Microdeletion/Microduplication Quantitative Fluorescent PCR (MQF-PCR). We used two very common microdeletion syndromes, the Williams-Beuren syndrome (WBS); (OMIM #194050) and the 22q11.2 microdeletion syndromes [Velocardiofacial syndrome (VCF); (OMIM #192430) and Di George syndrome (OMIM #188400)] to demonstrate the reliability and cost-efficiency of MQF-PCR. Additionally, we demonstrate that the same principle can be reliably used for detection of two microduplication syndromes, by using as examples the Lubs syndrome, involving the *MECP2* gene on Xq28 chromosome (OMIM #300260) and the 17q11.2 microduplication, involving the *NEUROFIBROMIN 1* (*NF1*) gene (OMIM #613113). We propose that MQF-PCR is a very useful procedure for laboratory confirmation of the clinical diagnosis of microdeletion/microduplication syndromes.

## Materials and Methods

### Ethics Statement

For the purpose of the assay development we used human subjects previously diagnosed with known microdeletion syndromes at GENE - Núcleo de Genética Médica, Belo Horizonte, MG, Brazil. The participants did not provide written or verbal consent and no other formal documented measures were taken. Since no new samples were needed, we chose to avoid the need to contact patients by using anonymization. Thus, DNA samples previously used for diagnosis and stored in our clinic were anonymized and used in testing the new diagnostic procedure. Since the new tests uniformly confirmed the previous diagnoses, they had value by adding reliability to them. This study was approved by the Ethics Committee of Santa Casa de Misericórdia in Belo Horizonte.

### Patients

Genomic DNA from normal male and female control individuals, and individuals previously diagnosed with specific microdeletion or microduplication syndromes (Table 1) was isolated from buccal swabs using a salting-out protocol [20].

**Table 1:** MQF-PCR results for patients with microdeletion and microduplication syndromes.

Syndrome	Patient	Z (PZ)-statisticsscore [34]
Williams-Beuren	194	−4.57
	385	−5.04
	603	−5.33
	604	−5.29
	3105	−5.10
	4183	−4.68
	4282	−5.13
	7350	−3.11
	8184	−5.47
	9234	−5.38
	10498	−4.76
	11275	−4.78
	11469	−5.05
	12681	−5.55
Velocardiofacial	8432	(−2.87)
	8441	(−3.93)
	9146	(−1.37)
	10097	(−2.45)
	10127	(−1.93)
	10332	(−2.17)
	10460	(−3.03)
	11153	(−3.03)
Lubs (*MECP2* duplication)	15982A [27], [29]	6.56
	15982B [27], [29]	4.12
	17863A [28]	9.40
	17863B [28]	7.65
17q11.2 microduplication [30]	*NF1*dup1	11.51
	*NF1*dup2	11.27
	*NF1*dup3	10.89

We studied initially 14 patients with the Williams-Beuren syndrome who had confirmation of their clinical diagnoses by studies of loss of heterozygozity of four microsatellites located in the critical region of the 7q11.23 microdeletion syndrome (a CCTT tetranucleotide polymorphism in the *ELN* gene [21] and three CA repeat polymorphisms, in the *ELN* gene [22], in the *LIMK1* gene [23] and in *D7S1870* [24], respectively). The diagnosis of these patients was further confirmed by Real-Time PCR using a 122 bp amplicon for the *ELN* gene chr7∶73,474,747–73,474,868 and a 149 bp amplicon in the *LIMK1* gene (chr7∶73,530,004–73,530,152). One patient had the diagnosis confirmed by aCGH.

We studied next eight patients clinically diagnosed as having the VCF syndrome. Laboratory confirmation of the clinical diagnoses were obtained by loss of heterozygozity studies of three microsatellites located in the critical region of the 22q11.2 microdeletion syndrome (the three CA repeat polymorphisms *D22S264* [25] and *D22S941* and *D22S944* [26], respectively). Further molecular confirmation was obtained by Real-Time PCR using four different amplicons as described by Weksberg et al. [10]: a 101 bp amplicon for the *CAT4* gene (chr22∶21,384,130–21,384,230), 101 bp amplicon in the *PRODH* gene (chr22∶18,918,663–18,918,763), a 101 bp amplicon in the *PIK4CA* gene (chr22∶21,105,251–21,105,351), and a 100 bp amplicon in the *COMT* gene (chr22∶19,956,086–19,956,185).

DNA samples from four patients (two pairs of affected brothers) with Lubs syndrome (microduplication of *MECP2*) were used [27]–[29]. DNAs from three patients with 17q11.2 microduplication, involving the Neurofibromatosis type 1 deletion syndrome critical region (OMIM #162200) diagnosed by aCGH and FISH, were a kind gift of Dr. Lisa G. Shaffer and Dr. Jill Mokry from the Signature Genomics Laboratories, Spokane, WA, USA [30]. To account for individual copy number variations, a reference DNA sample was created by pooling DNA from 100 male normal Brazilian individuals and used statistically to normalize results.

### MQF-PCR Assay Development

Minimal critical regions of each of the microdeletion syndromes (Table 1, GRCh37 human genome assembly) were used as queries for BLASTN (http://blast.ncbi.nlm.nih.gov) to identify regions with high sequence similarity that varied by 1–10 bp in length. In order to obtain maximum number of unique homologous regions, query sequences were split into 100–200 kb portions. Candidate pairs of highly homologous sequences were screened for 15–20 bp nucleotide stretches of absolute or highly conserved match representing potential detection primers. These were used as input for UCSC *In-Silico* PCR (http://genome.ucsc.edu/cgi-bin/hgPcr?command=start) to confirm amplification of the two potentially diagnostic sequences. Both coding and non-coding sequences were considered. Candidate pairs of gene regions and their corresponding homologous sequences were then screened for presence of known copy number variations in Database of Genomic Variants and SNPdb (http://projects.tcag.ca/variation; http://www.scandb.org). PCR primers (Table 2) were evaluated in Primer3 (http://frodo.wi.mit.edu) for optimal GC content, annealing temperature, and mispriming. Forward and reverse primers were tailed at the 5′ end using the M13-40 sequence to allow fluorescent labeling of amplicons with a NED-labeled M13-40 primer [31]. In order to ensure optimal fragment separation and reliable fluorescence signal detection, reverse primers were extended at 5′ end using a PIG-tail [32], which facilitates adenylation of the 3′ end of the forward strand. Degenerate primers were designed when necessary. For each syndrome studied a single pair of primers was selected to minimize cost.

**Table 2:** MQF-PCR primers used in detection of microdeletion and microduplication syndromes.

Syndrome	Primer name	Sequences (5′->3′)	Fragment sizes	Genomic locations (GRCh37)
Williams-Beuren	WBS-MQF-F	TGGGAGGGCCATTTTGTCAC^a^	chr.7: 218 bp	chr7: 73,536,635–73,536,831
	WBS-MQF-R	TTATTGTTCTGCRTCTGGG^b^	chr.18: 214 bp	chr18: 675,837–676,031
Velocardiofacial	VCF-MQF-F	GTATTTGGAAGWGTTTCTGTATAGA^a^	chr.22: 99 bp	chr22: 19,618,131–19,618,209
	VCF-MQF-R	GAGAACTGGGTTTACCTGAC^b^	chr.3: 104 bp	chr3: 88,205,817–88,205,900
Lubs (*MECP2* duplication)	LUBS-MQF-F	TGAAACCTGACTTGCTTCT^a^	chr.X: 197 bp	chrX: 153,276,119–153,276,295
	LUBS-MQF-R	GCACTGATTGTGGCAGAG^b^	chr.5: 198 bp	chr5: 32,649,017–32,649,192
17q11.2 microduplication	NF1-MQF-F	TGTTACCTGGTGTCTAGAGC^a^	chr.17: 159 bp	chr17: 29,468,898–29,469,033
	NF1-MQF-R	GCCCCTTAGACCATAATG^b^	chr.13: 153 bp	chr13: 19,673,801–19,673,931

a Universal M13-40 extension (5′-GTTTTCCCAGTCACGAC-3′) was added to the 5′ end of the primer to allow for cost-efficient fluorescent labelling of amplicons [31].

b A PIG-tail extension (5′-GTTTCTT-3′) was added to the 5′ end of the primer to promote full adenylation of the 3′ end of the forward strand [32].

### MQF-PCR Assay

PCR amplicons were labeled using a NED-labeled M13-40 universal fluorescent primer in a nested PCR to allow cost-efficient detection by fluorescent capillary electrophoresis [31]. PCR was carried out in total volume of 13 µl containing 7.9 µl 1x PCR buffer (30 mM TrisHCl pH 8.4, 75 mM KCl 2 mM MgCl_2_, 0.2 mM dNTPs), 0.3 µl fluorescently labeled M13-40-NED primer, 1 µl primer mix (0.1 µM forward primer, 1 µM reverse primer), 0.15 µl (0.75 U) Platinum *Taq* polymerase (Invitrogen), 3 µl water and 0.5 µl template DNA (5–300 ng/µl), using a stepdown PCR program: 95°C for 5 min, 95°C for 45 s, 60°C for 45 s, 72°C for 45 s, followed by 1°C stepdown for 9 cycles; then 50°C for 45 s, 72°C for 45 s for a total of 28 cycles and terminated with final 5 min extension at 72°C. Amplicons were separated and fluorescence intensities recorded by electrophoresis on an ABI 3130 Genetic Analyzer in total volume of 10 µl (8.8 µl highly deionized Hi-Di™ formamide with 0.2 µl GeneScan 500 LIZ size standard (ABI) and 1 µl PCR at 1∶10 dilution). Real-Time PCR verification was performed following the methods of Weksberg et al. [10], using the primers shown in Table S1. The LIMK1 primer, targeting an exon of *LIMK1* was designed using Primer3 and verified for amplification of single fragment by agarose gel electrophoresis. Loss of heterozygozity in WBS and VCF was assessed using polymorphic microsatellites (Table S1) as described in detail elsewhere [21]–[26].

### Data Analysis

Electrophoretic data were analyzed using GeneMapper 4.0 (ABI) and the test amplicon/control amplicon area ratio was calculated. In the polymerase chain reaction it is common for fragments of different sizes and/or in different genomic environments to present uneven amplification efficiencies. To correct for this, we used a normalization step, by dividing by the ratio of test amplicon/control amplicon area ratio by the analogous ratio of the reference pool of DNA, which was also processed in parallel.

The distribution of the normalized ratios was evaluated and tested for goodness of fit to a Gaussian distribution using the D’Agostino-Pearson test for normal distribution [33] in MedCalc v12 (MedCalc Software, Mariakerke, Belgium). If the ratios were symmetrical and conformed to a Gaussian distribution, they were converted to standard deviation Z scores. If the Gaussian distribution was rejected because of asymmetry, pseudo-Z (PZ) scores were calculated as described by Lanzante [34]. The overall performance of the MQF-PCR primer pairs for the detection of the microdeletion or microduplication syndrome was assessed using Receiver Operating Characteristic (ROC) analysis [35], [36] of the Z or PZ score statistics using MedCalc. Box-and-Whisker plot were also constructed [37] using MedCalc. Probability estimates of true positives, false positives, true negatives and false negatives [38], [39] were calculated using the Clinical Calculator 1 from the VassarStats website for statistical computation (http://vassarstats.net/clin1.html).

## Results

### Studies on the Williams–Beuren Syndrome (OMIM #194050)

**Figure 1:**
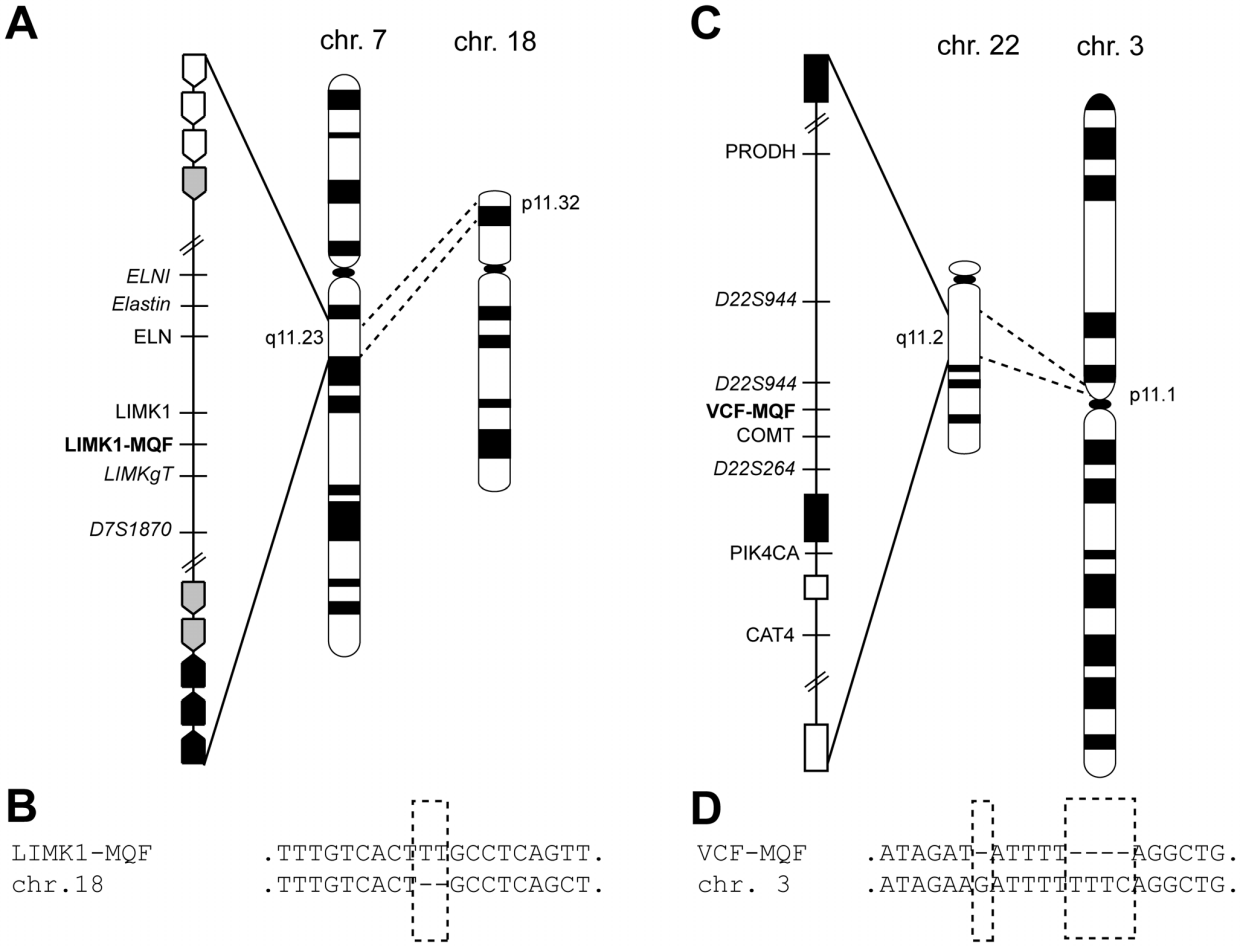
Schematic overview of MQF-PCR primer selection for Williams-Beuren and Velocardiofacial syndromes critical regions (not drawn to scale). A) Williams-Beuren critical region at 7q11.23 contains centromeric (white arrow), middle (grey arrow), and telomeric (black arrow) blocks of low copy repeats. The minimal critical region in this study represents the region that is flanked by the inner block of low copy repeats. Sequence similarity search identified a region that can be amplified using the LIMK1-MQF primers (bold) and has significant similarity to homologous region at 18p11.32. B) Multiple sequence alignment of the LIMK1-MQF region with its homologous region on chromosome 18. A deletion of 2 bp (dashed box) differentiates the two fragments amplified using the same primer pair. C) Velocardiofacial critical region at 22q11.2 is delimited by 4 blocks of low copy repeats. The minimal critical region in this study represents the region that is flanked by two blocks of low copy repeats most proximal to the centromere (black boxes). Sequence similarity search identified a region within the critical region that can be amplified using the VCF-MQF primers (bold) with significant similarity to a homologous region at 3p11.1. D) Multiple sequence alignment of the VCF-MQF region with its homologous region on chromosome 3. A deletion of 5 bp (dashed box) differentiates the two fragments amplified using the same primer pair. Microsatellite markers (italicized) and Real-Time PCR primers used in molecular diagnosis of patients are shown in both panels.

**Figure 2:**
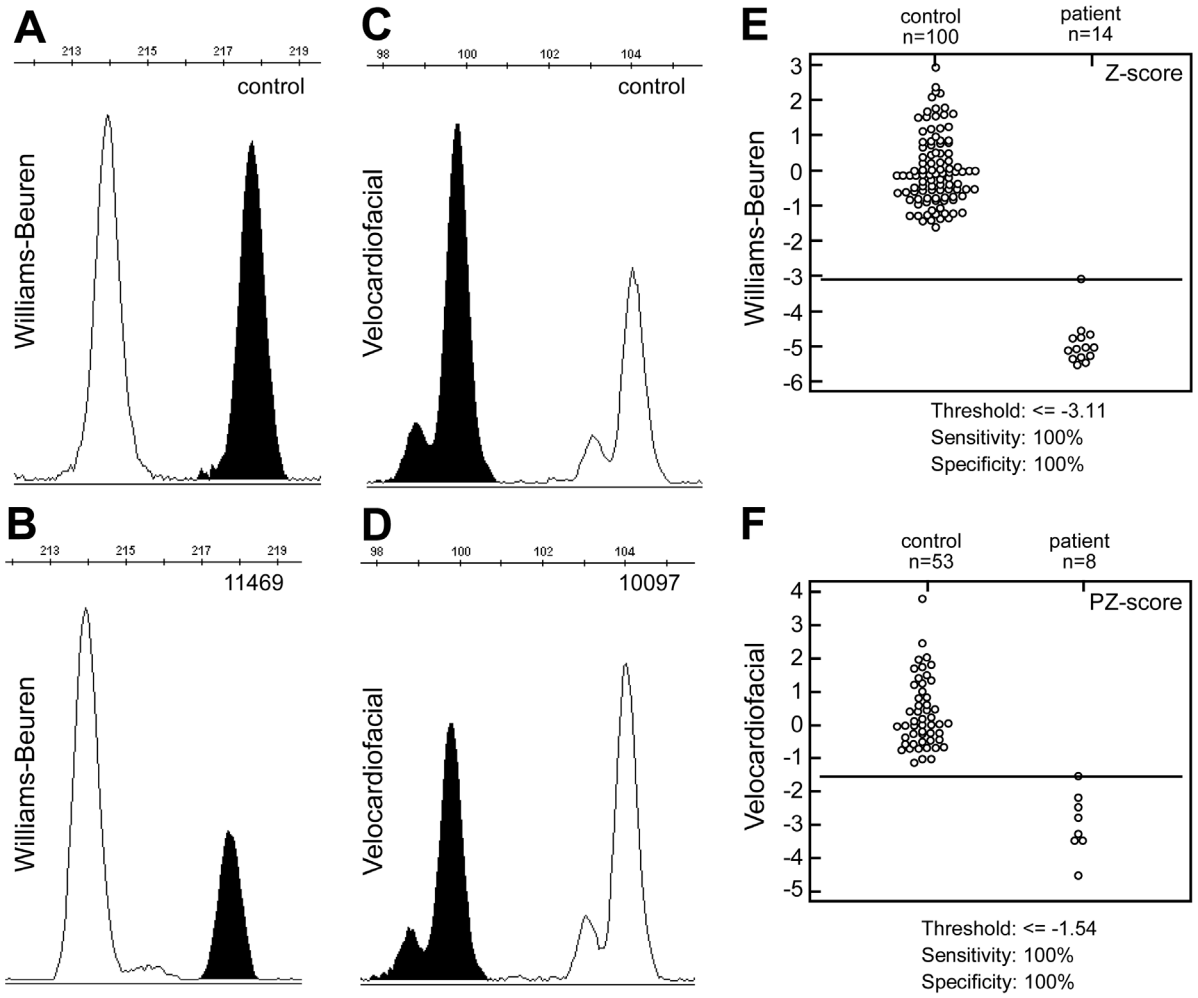
Evaluation of MQF-PCR in detection of Williams-Beuren and Velocardiofacial syndromes. A–D) Representative electropherograms showing the peak areas corresponding to the syndrome-related chromosomes (black) that are reduced by about 50% in comparison with the peaks representing the control chromosomes (white) between controls and affected individuals. Electropherogram depicting change in peak area between chromosome 7 and its control chromosome in normal (A) and individual with WBS syndrome (B). Electropherogram depicting change of the peak area between chromosome 22 and its control chromosome in normal (C) and individual with VCF syndrome (D). E–F) Interactive dot diagrams of ROC curve analysis of Z scores in WBS (E), and PZ scores in VCF syndrome (F). Both diagnostic primers achieved 100% diagnostic sensitivity and 100% diagnostic specificity. The number of cases analyzed and the detection threshold values for both syndromes are given.

To evaluate the facility of design and efficiency of our proposed MQF-PCR procedure, we first studied 14 cases of the Williams-Beuren syndrome, all previously diagnosed clinically in our clinical genetics service. Figure 1 shows an overview of the MQF-PCR strategy used for detection of the Williams-Beuren syndrome microdeletion. The WBS critical region, located at 7q11.23 (Figure 1A) contains several genes which, when in single copy, contribute to the phenotype of the syndrome [40]. We screened as a candidate the *LIMK1* gene, which is deleted in all WBS patients including atypical cases [41]. One segment (chr7∶73,536,635–73,536,831) showed homology to a region on chromosome 18p11.32 (chr18∶675,837–676,031). Both *LIMK1* and its homologous region on chromosome 18 could be amplified using the LIMK1-MQF primer pair (Table 2). On PCR, two amplicons were observed, one with a size of 214 bp (originating from the chromosome 18 homologue) and another, 4 bp longer, originating from the WBS critical region. Since WBS deletion patients were hemizygous, having only half of the starting amount of template DNA in comparison with the control region in chromosome 18, we expected a corresponding 50% reduction in PCR amplification compared with a normal individual. Figure 2A–B shows an electropherogram depicting change of peak area between a region on chromosome 7q11.23 (shown in black) and its control chromosome (white) in normal (Figure 2A) and the patient 11469 (Figure 2B). Indeed, the peak area ratio between the control and the WBS patient is reduced by about 50%. We then evaluated the detection performance of the MQF primers for the Williams-Beuren microdeletion using ROC curve analysis (Figure 2E). Using the Z-score values [34] the diagnostic primer achieved 100% sensitivity and 100% specificity. The Z-scores for all diagnosed patients are summarized in Table 1. The probabilities of a true positive result (positive predictive value) were estimated at 100% (95% confidence interval = 72–100%), a false positive result at 0% (95% confidence interval = 0–28%), a true negative result (negative predictive value) at 100% (95% confidence interval = 95–100%), and a false negative result at 0% (95% confidence interval = 0–5%).

### Studies on the Velocardiofacial Syndrome (OMIM #192430)

Having had excellent results with the Williams-Beuren syndrome, we then moved on to additionally study the very common microdeletion syndrome, Velocardiofacial syndrome, that results from hemizygosity for 22q11 [42]. We studied eight patients clinically diagnosed as having the VCF syndrome. The VCF critical region (Figure 2B) at position 22q11.2 is delimited by blocks of low copy repeats (LCRs). The vast majority of observed typical deletions share a common deleted region flanked by two LCRs most proximal to centromere (black boxes in Figure 1B), referred to by Shaikh et al. [42] as Block A and Block B. Within this region, a candidate sequence (chr22∶19,618,131–19,618,209) about 120 kb upstream of *TBX1* showed significant similarity to a homologous region at 3p11.1 (chr3∶88,205,817–88,205,900). Amplification of these two regions using the VCF-MQF primer that we designed (Table 2) resulted in two amplicons with size 99 bp (originating in chromosome 22) and 104 bp (originating in chromosome 3), differing by 5 bp in size. As an example, the change of peak area ratios between a region on chromosome 22q11.2 (shown in black) and its control chromosome (white) in a VCF patient and a normal individual is shown in Figure 2C–D. Similarly to the case with the Williams-Beuren microdeletion, the peak area ratio between the control (Figure 2C) and the VCF patient (Figure 2D) is reduced by about 50%. The detection performance of the primers for the 22q11.2 microdeletion was evaluated using ROC curve analysis. The normalized ratios in controls did not conform to Gaussian distribution, and thus the pseudo-Z score was calculated [34]. Using the PZ-score values the diagnostic primer achieved 100% sensitivity and 100% specificity (Figure 2F). The PZ-scores for all diagnosed patients are summarized in Table 1. The probabilities of a true positive result (positive predictive value) were estimated at 100% (95% confidence interval = 60–100%), a false positive result at 0% (95% confidence interval = 0–40%), a true negative result (negative predictive value) at 100% (95% confidence interval = 92–100%), and a false negative result at 0% (95% confidence interval = 0–8%). These results further confirm that MQF-PCR presents a reliable method for the detection of human microdeletion syndromes.

### Studies on Microduplication Syndromes

**Figure 3:**
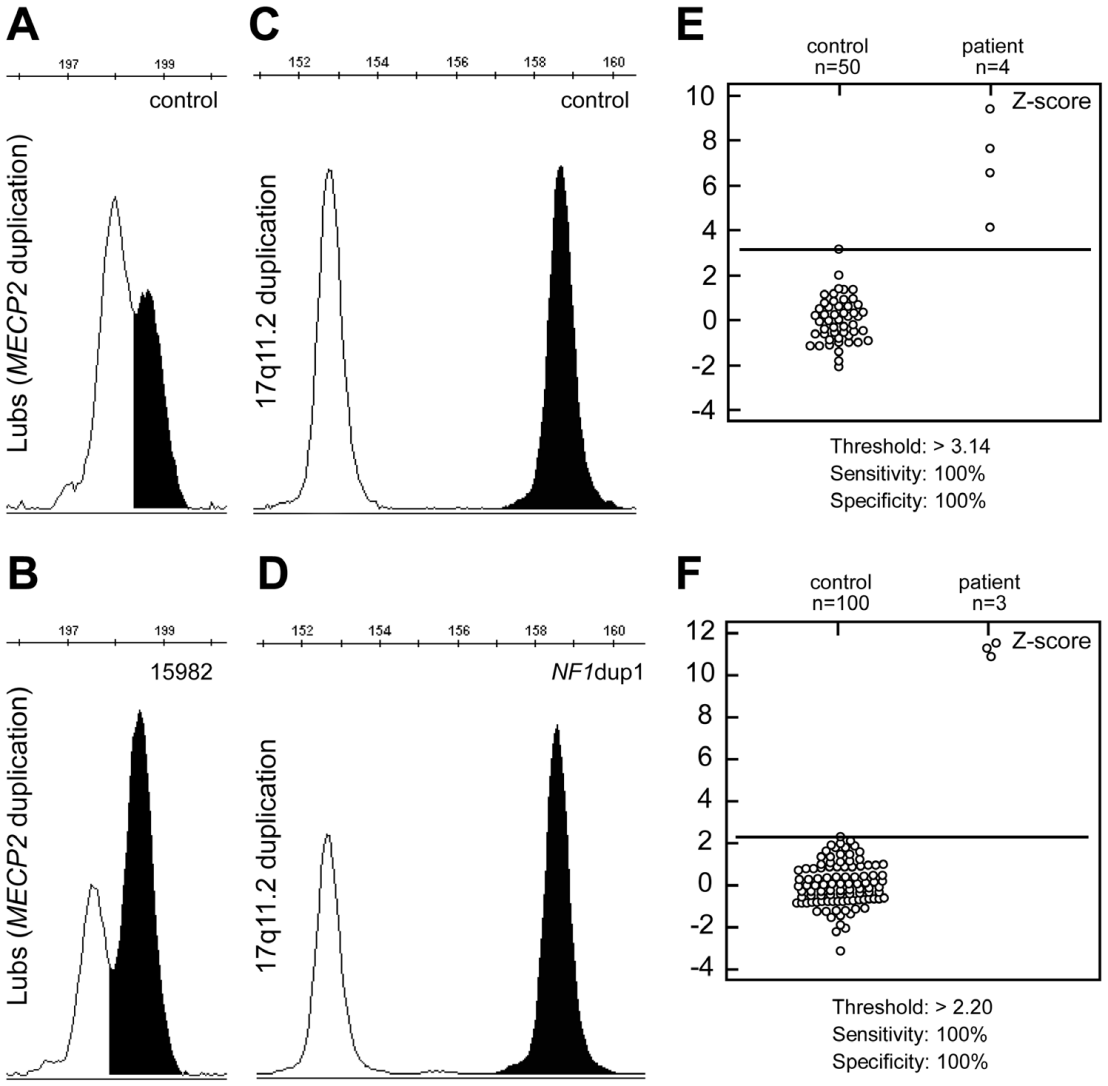
Detection of the Lubs (*MECP2* duplication) and 17q11.2 microduplication syndromes. Representative electropherograms showing changes in the peak area ratios between a control sample (A) and a patient (B) in diagnosis of the Lubs syndrome. The peak area corresponding to the duplicated region on chromosome Xq28 (black) has significantly increased in size compared to its control region on chromosome 5p13.3 (white). Electropherogram depicting change of the peak area between chromosome 17 and its control chromosome in normal (C) and individual with microduplication of 17q11.2 (D) The peak area corresponding to the duplicated region on chromosome 17 (black) has significantly increased in size compared to its control region on chromosome 13q12.11 (white). E–F) Interactive dot diagrams of ROC curve analysis of Z scores in Lubs (E), and 17q11.2 microduplication syndrome (F). Both diagnostic primers achieved 100% diagnostic sensitivity and 100% diagnostic specificity. The number of cases analyzed and the detection threshold values for both syndromes are given.

To test the efficiency of MQF-PCR in the detection of microduplication events, we studied the X-linked microduplication syndrome at Xq28 (Lubs syndrome; OMIM#300260) and an autosomal duplication involving the gene *NF1*. For the former, we scanned the *MECP2* duplication critical region [43] and identified a sequence (chrX:153,276,119–153,276,295) that shared high homology with a sequence on chromosome 5p13.3 (chr5∶32,649,017–32,649,192). PCR amplification using the same primer pair (Table 2) yielded two fragments that differed by 1 bp in size. Two patients, previously described [29]–[31] were studied, and one affected brother of each was also analyzed. Figure 3A–B shows an electropherogram illustrating changes in peak area ratio between control (Figure 3A) and a patient known to have the *MECP2* microduplication (Figure 3B). The area of the peak corresponding to chromosome Xp28 (black) had approximately doubled compared to the control peak area (white). Similar results were obtained for the other 3 patients known to have the *MECP2* microduplication syndrome. The detection performance of the primers for the Lubs syndrome was evaluated using ROC curve analysis. Using the Z-score values [34] summarized in Table 1, the diagnostic primer achieved 100% sensitivity and 100% specificity (Figure 3E). The probabilities of a true positive result (positive predictive value) were estimated at 100% (95% confidence interval = 40–100%), a false positive result at 0% (95% confidence interval = 0–60%), a true negative result (negative predictive value) at 100% (95% confidence interval = 91–100%), and a false negative result at 0% (95% confidence interval = 0–9%).

For the autosomal duplication involving the gene *NF1* we designed a diagnostic primer in the critical region at 17q11.2 (chr17∶29,468,898–29,469,033) that shared high homology with a sequence on chromosome 13q12.11 (chr13∶19,673,801–19,673,931). PCR amplification using the same primer pair (Table 2) yielded two fragments that differed by 6 bp in size. We studied three patients previously diagnosed by FISH and aCGH with microduplication of *NF1* (OMIM #613113) [30]. Figure 3C–D shows an electropherogram illustrating changes in peak area ratio between control (Figure 3C) and a patient known to have the 17q11.2 microduplication (Figure 3D). The area of the peak corresponding to chromosome 17q11.2 (black) had increased by about 30% compared to the control peak area (white). We observed similar results with the other two patients. The detection performance of the primers for the 17q11.2 microduplication was evaluated using ROC curve analysis. Using the Z-score values [34] summarized in Table 1, the diagnostic primer achieved 100% sensitivity and 100% specificity (Figure 3F). The probabilities of a true positive result (positive predictive value) were estimated at 100% (95% confidence interval = 31–100%), a false positive result at 0% (95% confidence interval = 0–69%), a true negative result (negative predictive value) at 100% (95% confidence interval = 95–100%), and a false negative result at 0% (95% confidence interval = 0–5%). The results from detection of the Lubs syndrome and the 17q11.2 microduplication further confirm that MQF-PCR also presents a reliable method for the detection of human microduplication syndromes.

## Discussion

In the past few years, dozens of clinically relevant chromosomal microdeletions and microduplications have been described in humans, often associated with mental deficiency, autism and/or physical malformations [1]. Since these small genomic arrangements are generally below the detection limit of optical microscopy, it is essential to use molecular diagnostic procedures to furnish an explanation for the observed symptoms and signs and to provide clinical and genetic prognoses for the patients and their families. In developed countries, pangenomic microarray-based molecular tests, particularly aCGH, have become the gold standard for such laboratory diagnosis [44]. However, these tests are very costly and depend on availability of expensive equipment that needs to be frequently upgraded. As a result of these high costs, patients in developing countries do not have access to the tests and are frequently left undiagnosed, with great prejudice for their families.

Although some microdeletion/microduplication syndromes have ill-defined phenotypes, many of them, indeed the most common ones, can be reliably diagnosed on clinical grounds. This propitiates laboratory confirmation to be made using simpler targeted molecular tests. In this article we describe the use of an inexpensive quantitative fluorescent PCR method for targeted identification of patients bearing microdeletion and microduplication syndromes that we proposed to call MQF-PCR (Microdeletion/Microduplication Quantitative Fluorescent PCR). The MQF-PCR method achieved 100% sensitivity and 100% specificity in the detection of patients with the Williams-Beuren syndrome, Velocardiofacial syndrome, Lubs syndrome (*MECP2* duplication) and the 17q11.2 microduplication involving the gene *NF1*. We believe that our method can be expanded to any syndrome originating from loss or gain or genomic material. We developed highly promising primer pairs for several other rarer syndromes where comprehensive validation is pending the collection of additional patients.

The MQF-PCR test is very rapid, allowing results to be reached in a just a few hours. Since the method uses a very cost-efficient fluorescent labeling utilizing a single universal fluorescent primer [31] coupled with signal detection provided using equipment readily available in paternity and forensic laboratories in developing countries, the price of the necessary materials is low. In fact, it is possible to perform complete analyses at less than US$8.00 per sample, significantly less than a comparable targeted test using MLPA, which costs US$100.00 per sample in Brazil. Unlike Real-Time PCR, our method employs a single primer that allows robust internal control of amplification throughout the PCR reaction. Any deviations between amplification efficiency of the test and control amplicons are therefore tightly controlled and result in excellent reproducibility. Consequently, only one diagnostic primer pair was necessary to accurately identify affected individuals, which also contributes to reduced cost compared to Real-Time PCR and MLPA where multiple primer pairs are used in order to gain confidence in results. Our results were reliable and indeed we found no advantage of performing experiments in replicates. This probably was a consequence of normalization of our results with those of a DNA pool of one hundred normal individuals amplified and analyzed in parallel with the test sample. Furthermore, by the fact that it makes use of ideally matched internal controls, it delivers satisfactory results using a range of template DNA concentrations and is not limited to high quality DNA. The overall template quality does not seem to affect the performance of our method. Indeed, samples stored for 10 years at 4°C have provided satisfactory results. MQF-PCR has shown to be applicable to clinical material embedded in paraffin.

In summary, MQF-PCR has been developed as a rapid, reliable and cost-effective method for screening and detection of several human microdeletion and microduplication syndromes. Its ease and low cost make it accessible to any clinical diagnostic laboratory. It is thus ideally suited for diagnostic use in developing countries, but has general applicability as well. However, its validation as a conclusive diagnostic procedure is still pending the collection of additional patients.

## Supporting Information

Table S1 **Summary of primers used in molecular diagnosis confirmation of Williams-Beuren and Velocardiofacial syndromes using polymorphic microsatellites and Real-Time PCR.**
(DOCX)
