# Supplementary material for: Simple, Rapid and Inexpensive Quantitative Fluorescent PCR Method for Detection of Microdeletion and Microduplication Syndromes
Source: PLoS One. 2013 Apr 19;8(4):e61328. doi: 10.1371/journal.pone.0061328 (PMC3631209; doi:10.1371/journal.pone.0061328)
Supplement: Table S1 — Summary of primers used in molecular diagnosis confirmation of Williams-Beuren and Velocardiofacial syndromes using polymorphic microsatellites and Real-Time PCR. (DOCX) [file pone.0061328.s001.docx]

| **Syndrome** | **Purpose** | **Primer name** | **Sequence (5’->3’)** | **Primer concentration (µM)** |
| --- | --- | --- | --- | --- |
| Williams-Beuren | Microsatellite assay | LIMKgT [23] | F-TGCCATCACTCTACTGCAGCC | 0.02 |
|  |  |  | R-CATTAAGATTCAGATGGTCC | 0.02 |
|  |  | Elastin [22] | F-ATGAGACGTGGTCAAGGGTAT | 0.02 |
|  |  |  | R-GGGATCCCAGGTGCTGCGGTT | 0.02 |
|  |  | D7S1870 [24] | F-TTCACTCAGGAAGTGGC | 0.02 |
|  |  |  | R-TGGTGATGTGCTTTACTACG | 0.02 |
|  |  | ELNI [21] | F-GCCCACATGGGCAGATTGCT | 0.02 |
|  |  |  | R-CCCTCATCCACAGACAGGTC | 0.02 |
|  | Real-Time PCR | LIMK1^a^ | F-GCCAATACAGCGTATCAGAGGT | 0.4 |
|  |  |  | R-AGCCACCACCACATTCTTGT | 0.4 |
|  |  | ELN [S1] | F-GTTGGTGTCGGCGTCCC | 0.3 |
|  |  |  | R-TCAGGGGACAGGCTCCG | 0.3 |
|  |  | HMBS^b^ [S1] | F-ACGGCTCAGATAGCATACAAG | 0.1 |
|  |  |  | R-ATGCCTACCAACTGTGGGTCA | 0.1 |
| Velocardiofacial | Microsatellite assay | D22S264 [25] | F-ATTAACTCATAAAGGAGCCC | 0.01 |
|  |  |  | R-CACCCCACCAGAGGTATTCC | 0.01 |
|  |  | D22S941 [26] | F-CAGGTTACAAAGTACATTAACTT | 0.02 |
|  |  |  | R-CAAGAAATGGTTGGAGCTGGT | 0.02 |
|  |  | D22S944 [26] | F-CATGTGAAAGATGCTACTTCC | 0.01 |
|  |  |  | R-ATCCCATGCTCCTCCCCAT | 0.01 |
|  | Real-Time PCR | PRODH [10] | F-GGGAAAGGAGAGTTCAGGCAG | 0.2 |
|  |  |  | R-GCTTGTTGAATAGCCTCTGTCCTAG | 0.2 |
|  |  | COMT [10] | F-GTGCTACTGGCTGACAACGTGAT | 0.2 |
|  |  |  | R-GGAACGATTGGTAGTGTGTGCA | 0.2 |
|  |  | PIK4CA [10] | F-ATGCTTGTGCGACGCAGAC | 0.2 |
|  |  |  | R-CCTCAGCCATGTTGACTCAGC | 0.2 |
|  |  | CAT4 [10] | F-TACCTGGGCTTCTTGGATGG | 0.2 |
|  |  |  | R-AAGACAAGCACGCAGCCTATG | 0.2 |
|  |  | HEM3^b^ [10] | F-TGCACGGCAGCTTAACGAT | 0.2 |
|  |  |  | R-AGGCAAGGCAGTCATCAAGG | 0.2 |

^a^ GENE-Núcleo de Genética Médica, MG, Brazil

^b^ internal reference primer

**Supporting References**

S1 Saugier-Veber P, Goldenberg A, Drouin-Garraud V, de La Rochebrochard C, Layet V, et al. (2006) Simple detection of genomic microdeletions and microduplications using QMPSF in patients with idiopathic mental retardation. Eur J Hum Genet 14: 1009-1017.
